# Erk1/2 Mediates Leptin Receptor Signaling in the Ventral Tegmental Area

**DOI:** 10.1371/journal.pone.0027180

**Published:** 2011-11-04

**Authors:** Richard Trinko, Geliang Gan, Xiao-Bing Gao, Robert M. Sears, Douglas J. Guarnieri, Ralph J. DiLeone

**Affiliations:** 1 Division of Molecular Psychiatry, Ribicoff Research Facilities, Department of Psychiatry, Yale University School of Medicine, New Haven, Connecticut, United States of America; 2 Department of Obstetrics and Gynecology, Yale University School of Medicine, New Haven, Connecticut, United States of America; University of South Florida College of Medicine, United States of America

## Abstract

Leptin acts on the ventral tegmental area (VTA) to modulate neuronal function and feeding behavior in rats and mice. To identify the intracellular effectors of the leptin receptor (Lepr), downstream signal transduction events were assessed for regulation by direct leptin infusion. Phosphorylated signal transducer and activator of transcription 3 (pSTAT3) and phosphorylated extracellular signal-regulated kinase-1 and -2 (pERK1/2) were increased in the VTA while phospho-AKT (pAKT) was unaffected. Pretreatment of brain slices with the mitogen-activated protein kinase kinase -1 and -2 (MEK1/2) inhibitor U0126 blocked the leptin-mediated decrease in firing frequency of VTA dopamine neurons. The anorexigenic effects of VTA-administered leptin were also blocked by pretreatment with U0126, which effectively blocked phosphorylation of ERK1/2 but not STAT3. These data demonstrate that pERK1/2 may have a critical role in mediating both the electrophysiogical and behavioral effects of leptin receptor signaling in the VTA.

## Introduction

Leptin is a protein hormone produced by adipocytes that serves to communicate fat levels to the brain. Within the central nervous system (CNS), leptin acts on multiple brain regions including the brainstem, hypothalamus, hippocampus, and ventral tegmental area (VTA) by activating the cytokine type I leptin receptor [1], [2], [3], [4], [5]. Work on the hypothalamus has shown that Lepr signaling regulates multiple downstream pathways to modulate neuronal function, food intake, and body weight homeostasis. Lepr is coupled to Janus Kinase 2 (JAK2), which is required for all leptin-mediated signaling, including recruitment and subsequent activation of STAT3, ERK1/2, and phosphatidylinositol-3-kinase (PI3-K) [6]. Conditional mutant mice lacking either neural STAT3 expression, or STAT3 activation, have recapitulated the obese phenotypes observed in leptin deficient (*ob/ob*) and the Lepr deficient (*db/db*) mice [7], [8], [9]. While these mutant models suggest that STAT3 is clearly an important mediator of leptin signaling, the rapid regulation of neuronal firing in the hypothalamus [10], and in the VTA [4], is not likely to be mediated by STAT3-dependent transcriptional events.

In the hypothalamus, Lepr signaling can also activate ERK1/2 and PI3-K [11], [12], [13], [14]. Pretreatment of rats with MEK1/2 inhibitors blocked leptin-induced ERK1/2 phosphorylation in the hypothalamus, as well as attenuating the homeostatic feeding effects of Lepr [15]. It has been demonstrated that PI3-K is also required for the anorexic effects of insulin as well as leptin in the CNS, thus illustrating potential cross-talk between leptin and insulin signaling [14], [16]. Moreover, in the hypothalamus, it has been suggested that the ERK1/2 pathway mediates leptin-induced reduction of firing rates, while PI3-kinase is responsible for the leptin's disinhibitory effects on firing [17].

In the VTA, Lepr signaling reduces dopamine neuron firing and food intake, while RNAi-mediated knockdown of Lepr in the VTA results in a chronic increase in food intake without an associated weight gain [4]. These data, complemented with studies by others [5], supports a physiologic role of leptin signaling to this brain region. Like the hypothalamus, leptin signaling results in STAT3 phosphorylation at residue Tyr-705 in the VTA [4]. In contrast to the hypothalamus however, PI3-kinase appears not to be a mediator of leptin's effects in the VTA [18]. This represents the first observed difference in leptin signaling pathways between these brain regions, thus highlighting the need for additional characterization. In the VTA, it remains unclear whether the ERK1/2 pathway: 1) is regulated by leptin, 2) contributes to dopamine neuron firing, and 3) has a role in mediating leptin feeding responses specific to this brain region. Here, we evaluate potentially important Lepr signaling events using western blotting, electrophysiology and behavioral pharmacology to identify the contributions of the ERK1/2 pathway in the VTA.

## Results

### Direct leptin infusion to the VTA of rats results in multiple phosphorylation events

**Figure 1:**
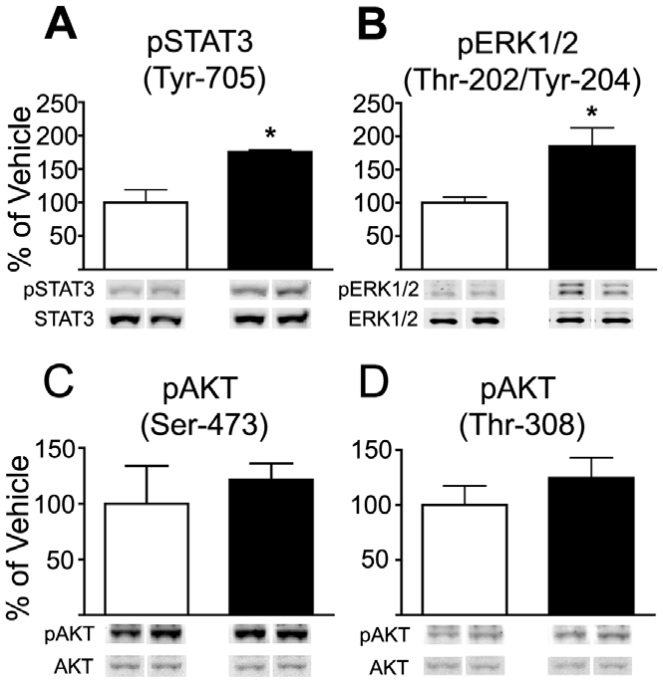
Direct leptin to the rat VTA induced phosphorylation events. Regulation of various Lepr pathways was evaluated 45 minutes after direct leptin infusion to the VTA of cannulated awake adult male rats. Ratios of phospho-/total signal were used to calculate the percent change from control tissue. Blots shown are representative. Open bars represent vehicle while black bars represent leptin treatment. ***A.*** pSTAT3 (Tyr-705) (*n = 4*) *, (*P = 0.0077*). ***B.*** pERK1/2 (Thr-202, Tyr-204) (*n = 4*) *, (*P = 0.0252*). ***C.*** pAKT (Ser-473) (*n = 4*). ***D.*** pAKT (Thr-308) (*n = 4*).

To assess Lepr signaling pathways in the VTA, leptin or vehicle was directly infused to the VTA of rats, which were sacrificed 45 minutes later. Western blot analysis of dissected VTA tissue revealed phosphorylation events within two canonical leptin signaling pathways. Consistent with our prior study, leptin induced the phosphorylation of STAT3 (Tyr-705), as well as pERK1/2 (Thr-202, Tyr-204) (Fig. 1A, B). The PI3-K pathway was evaluated indirectly by measuring a downstream target, AKT. In contrast with pSTAT3 and pERK1/2, neither of the two residues known to be involved in regulating AKT activity (Thr-308, Ser-473) were affected by leptin at this time point (Fig. 1C, D).

### U0126 abolishes the firing response of dopamine neurons to leptin in the VTA of mice

**Figure 2:**
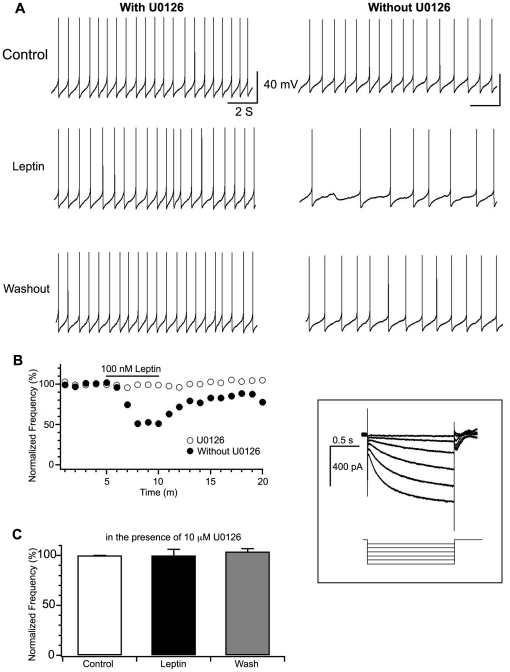
U0126 abolished the firing response of dopamine neurons to leptin in mice. See results section for statistical information. ***A.*** Representative traces of action potentials recorded before, during, and after the application of leptin to mouse VTA DA neurons in slices with (left panel) or without (right panel) U0126 (10 mM). ***Box***, H-current recorded in VTA DA neurons. Hyperpolarizing voltage steps from −50 to −120 mV for 2 s generates a large/h current (>100 pA), the presence of which identifies dopamine neurons. ***B.*** A representative time course of the response of action potential frequency to leptin in the presence (open symbol) or absence (solid symbol) of U0126 in VTA DA neurons. The solid horizontal black line indicates the duration of leptin application. ***C.*** A summary of normalized frequency of action potentials before, during, and after the application of leptin in DA neurons in VTA slices pre-treated with U0126.

The role of ERK1/2 in mediating the electrophysiological response of dopamine neurons to leptin was assessed by blocking the MEK1/2 kinase needed for phosphorylation and activation of ERK1/2. Coronal mouse slices containing the VTA were incubated with or without the MEK1/2 inhibitor U0126 prior to application of leptin. Spontaneous firing events of dopamine cells were observed by whole-cell current clamp. Consistent with our prior study [4], application of leptin to the bath resulted in decreased frequency of action potentials (48.5±27.3% of control, n = 3) throughout the duration of application and returned to baseline level (102.7±8.6% of control, n = 3) after the removal of leptin. Representative traces are shown in Fig. 2A (right panel), and a representative timeline in Fig. 2B. This effect was completely attenuated when U0126 was applied for 20 minutes prior to leptin administration (Fig. 2A, left panel; Fig. 2B). The action potential frequency was 100.1±6.2% of control (P>0.05, n = 6, t-test) in the presence of leptin and U0126, and 104.2±3.4% of control (n = 6) after the removal of leptin, as summarized in Fig. 2C. Notably, there was no change in action potential frequency as a result of U0126 during pretreatment (Fig. 2C).

### U0126 abolishes the feeding effects of leptin signaling in the VTA of rats

**Figure 3:**
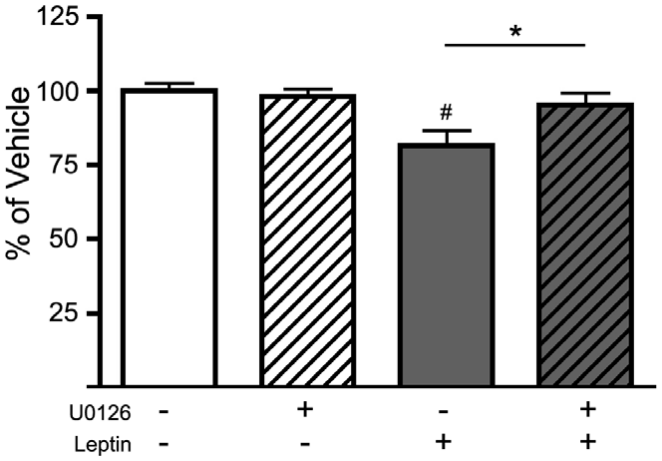
ERK1/2 mediates the anorexigenic effect of leptin in the VTA of rats. Direct leptin infusion to the VTA of rats caused a significant decrease in food intake, while pretreatment with U0126 ICV blocked this effect. Treatment groups include: vehicle^ICV^/vehicle^VTA^ (open bars; n = 13), U0126^ICV^/vehicle^VTA^ (open-striped bars; n = 11), vehicle^ICV^/Leptin^VTA^ (gray bars; n = 12), U0126^ICV^/Leptin^VTA^ (gray-striped bars; n = 13). * Represents significant effect of leptin (F_(1, 45)_ = 7.78, *P<0.008*). ^#^ Represents significant Leptin-U0126 interaction (F_(1, 45)_ = 4.17, *P<0.047*).

We have previously demonstrated that leptin infusions to the VTA results in consistent and robust reductions in intake [4]. To investigate the role of the ERK1/2 pathway in mediating the behavioral effects of leptin, U0126 was infused intracerebroventricular (ICV) 1.5–2 hours before leptin infusion in the VTA, and food intake was assessed. Rats in the U0126^ICV^/vehicle^VTA^ group exhibited no change in basal feeding over a 23 hour period when compared to the vehicle^ICV^/vehicle^VTA^ group (Fig. 3). Rats in the vehicle^ICV^/leptin^VTA^ group exhibited a significant decrease in food intake over 23 hours. Finally, rats in the U0126^ICV^/leptin^VTA^ group exhibited no changes in feeding, suggesting that ERK1/2 signaling is required for leptin's effects on feeding in the VTA.

### U0126 blocks leptin-induced phosphorylation of ERK1/2, but not STAT3 (Tyr-705) in the VTA of rats

**Figure 4:**
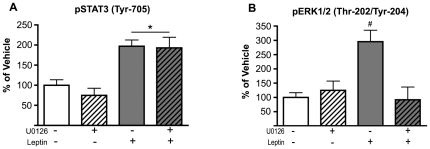
U0126 selectively blocks phosphorylation of the ERK1/2 pathway in the VTA of rats. Pretreatment of rats with U0126 prevented leptin-induced phosphorylation of ERK1/2 without affecting STAT3 phosphorylation. Treatment groups include: vehicle^ICV^/vehicle^VTA^ (open bars; n = 5), U0126^ICV^/vehicle^VTA^ (open-striped bars; n = 4), vehicle^ICV^/Leptin^VTA^ (gray bars; n = 4), U0126^ICV^/Leptin^VTA^ (gray-striped bars; n = 4). Ratios of phospho-/total signal were used to calculate the percent change from control tissue. ***A.*** Evaluation of pSTAT3 (Tyr-705) phosphorylation across groups. * Represents significant main effect of leptin (F_(1, 15)_ = 41.9, *P<0.001*). ***B.*** Evaluation of ERK1/2 (Thr-202, Tyr-204) phosphorylation across groups. ^#^ Represents significant interaction of Leptin-U0126 (F_(1, 15)_ = 14.4, *P<0.002*); significant leptin effect (F_(1, 15)_ = 7.9, *P<0.013*); significant U0126 effect (F_(1, 15)_ = 9.3, *P<0.008*).

It is possible that the U0126 compound was interfering with leptin receptor activation of STAT3 in response to leptin. To test this, pSTAT3 (Tyr-705) was assessed in the presence of the Mek inhibitor. U0126 was infused 1.5–2 hours before leptin infusion into the VTA, and the VTA was dissected 45 minutes later to evaluate pSTAT3. VTA pSTAT3 (Tyr-705) levels in U0126^ICV^/vehicle^VTA^ rats remained unchanged relative to vehicle^ICV^/vehicle^VTA^ animals, while pSTAT3 (Tyr-705) in both vehicle^ICV^/leptin^VTA^ and U0126^ICV^/leptin^VTA^ rats showed a significant increase (Fig. 4A). This demonstrates that the behavioral and electrophysiological effects of U0126 are not due to indirect effects via STAT3 phosphorylation. In contrast with pSTAT3, VTA pERK1/2 (Thr-202, Tyr-204) levels were found to be unchanged in U0126^ICV^/leptin^VTA^ rats (Fig. 4B), indicating effective blockade of the MEK-ERK pathway.

## Discussion

Lepr signaling studies in the hypothalamus have identified key components needed for leptin signaling [19], [20], [21], [22], [23]. Our previous work identified a physiologic role for Lepr expression in the VTA [4]. Direct infusion of leptin to the VTA of naïve rats resulted in hypophagia, while local knockdown of Lepr resulted in hyperphagia, thereby demonstrating responses to localized exogenous, as well as endogenous leptin [4]. The identification of insulin and leptin receptor coexpression on dopamine neurons in the VTA [13], and preliminary signaling studies [4], [5] suggest similarities in pathway activities between the VTA and hypothalamus. Here, we initially examined these signaling events by evaluating protein phosphorylation in the VTA after direct leptin infusion. ERK1/2 is regulated by leptin, and blockade of this pathway eliminated leptin's effects on both neuronal firing and feeding behavior.

Consistent with published data, pSTAT3 (Tyr-705) was increased in the VTA after direct leptin infusion [4], [5]. ERK1/2 also exhibited increased phosphorylation in the VTA after direct leptin. These findings highlight a similarity in leptin signaling between the hypothalamus and the VTA. In contrast, both regulatory residues on AKT, a known downstream target of insulin-induced PI3-K activity [24], showed no significant change after direct leptin. The lack of PI3-K regulation is consistent with work showing no role of this pathway in mediating the effects of leptin in the VTA [18]. This illustrates a potential divergence in leptin signaling between brain regions, as it has been previously suggested that hypothalamic Lepr activation of PI3-K is crucial for mediating the feeding effects of leptin [16], [18], [25], [26].

To date, there is little research evaluating the role of ERK1/2 in mediating the electrophysiological effects of leptin. It has been demonstrated that leptin can regulate calcium concentrations in isolated hypothalamic neuropeptide Y (NPY) and proopiomelanocortin (POMC) neurons in an opposing manner [27]. More recently, Wang and colleagues suggested a role for hypothalamic ERK1/2 in mediating leptin's effect on calcium regulation in NPY neurons, but not POMC neurons [17]. In the VTA, leptin reduces dopamine firing frequency [4] and data presented here suggests that ERK1/2 mediates this effect in the VTA dopamine neurons, as with NPY neurons in the hypothalamus [17].

To evaluate the behavioral relevance of the biochemical and electrophysiological findings, ERK1/2 was tested for its role in mediating leptin's anorectic effects. Rats treated with leptin to the VTA exhibited a reduction in food intake, consistent with our previous data. Pretreating rats with U0126 attenuated this effect, thus suggesting a role for ERK1/2 as a critical component of Lepr signaling in the VTA. The similarity with our findings and those of Rahmouni and colleagues, with the hypothalamus, is notable. In both cases, treatment with inhibitors alone did not alter basal feeding, however, they did attenuate effects of leptin. Additionally, both studies demonstrate successful pharmacological blocking of ERK1/2 phosphorylation, while pSTAT3 (Tyr-705) levels remained high. It is important to note that a major difference between our feeding study and the Rahmouni study involves site-specific delivery of leptin. Rahmouni and colleagues delivered both the inhibitors and leptin ICV, thus potentially affecting multiple feeding circuits in the CNS, including the VTA, which could theoretically contribute to their behavioral findings. In our study, we delivered leptin directly to the VTA, thus attempting to localize its effects and to identify the importance of ERK1/2 signaling within this region.

The observation that leptin increased pSTAT3 (Tyr-705) despite pretreatment with U0126 suggests that STAT3 may not be sufficient for the cellular and behavioral effects of leptin in the VTA. It is important to emphasize that pTyr-705 assessment may not always reflect STAT3 activity [28]. Moreover, even if STAT3 activation is not sufficient for the rapid effects of leptin, it is still possible that long-term modulation of neuronal function is mediated by transcriptional changes downstream of STAT3. Conversely, other transcription factors may be regulated by ERK1/2 to mediate leptin signaling in the VTA. It remains unknown if the rapid change in dopamine firing caused by leptin is responsible for the long-term feeding changes, or whether these are dissociable. However, it is notable that the data presented here suggest that ERK1/2 is important for the observed neuronal and behavioral effects. These findings have a potential impact on future research on behaviors regulated by leptin activity in the VTA. Further studies are required to determine any potential role of VTA STAT3, and to further characterize the downstream effectors that respond to ERK1/2 activation in dopamine neurons of the VTA.

## Materials and Methods

### Antibodies

The following antibodies were purchased from Cell Signaling Technology (Beverly, Ma.): phospho-STAT3 (Tyr-705), STAT3, phospho-ERK1/2 (Thr-202, Tyr203), phospho-AKT (Ser-473), phospho-AKT (Thr-308), and AKT. Pan ERK antibody was purchased from BD Biosciences (San Jose, Ca.).

### Animals

Animal experiments were done in accordance with Yale University School of Medicine and IACUC animal care guidelines. Sprague Dawley rats were purchased from Charles River Laboratories, and given access to ad libitum chow and water. Standard rat chow used was RMH-3000 from Lab Diet (Richmond, In.). Rats were housed in multiples prior to surgeries, after which they were singly housed. Mice used for electrophysiology were C57BL/6J (Jackson Labs, Bar Harbor, Maine). The environment was a controlled 12 hr light, 12 hr dark cycle.

### Rat Cannulation Surgery

Rat VTA cannulations consisted of double-barreled cannulae, while intracerebral ventricular (ICV) cannulations consisted of single barrel cannulae. All cannulae were purchased from Plastics1 (Roanoke, Va.). All stereotaxic coordinates are based on the standard rat atlas [29]. All animals weighed at least 300 g at the time of surgeries, and were anesthetized with Nembutal. The following coordinates were used: 1) VTA: anterior-posterior (A/P) from bregma −5.8 mm; dorsal-ventral (D/V) from skull surface −7.8 mm, 2) ICV: A/P from bregma −0.8 mm; D/V from dura −3.4 mm; and medial-lateral from midline −1.5 mm. All animals were single housed for the remainder of the experiment, and were allowed 1 week of recovery prior to subsequent manipulations.

### Infusions for Leptin Biochemistry

All infusions were carried out in cannulated animals, and occurred just prior to or shortly after the onset of the dark period. Mouse recombinant leptin (498-OB) was purchased from R&D Systems (Minneapolis, Mn). For direct administration to the VTA, 1.0 µg (0.5 µg on each side) of leptin or vehicle (1× PBS) in 0.5 µl, was infused over 2 minutes, after which the injectors remained in the cannulae for an additional minute before removal. Sacrifice by rapid decapitation occurred 45 minutes after infusion. For ICV/VTA infusions, ICV preceded VTA by 1.5 to 2 hours. 2.0 µg U0126 (Promega, Madison, Wi.) or vehicle (DMSO) in 3.0 µl was infused over 2 minutes ICV. VTA infusions were as described above, and animals were sacrificed by rapid decapitation 45 minutes afterward.

### Infusions for Ad Libitum Feeding Behavior

All infusions were to cannulated awake rats, and occurred just prior to or shortly after the onset of the dark period. Food was removed from each cage immediately before infusions. All volumes and concentrations for vehicles, U0126, and leptin were as described above. All ICV infusions preceded the VTA infusions by 1.5 to 2 hrs. After VTA infusions, animals were exposed to food, and intake was assessed.

### Brain Extraction Dissection

After decapitation, brains were quickly extracted and frozen on dry ice. Frozen 300 µm coronal sections were taken by cryostat and mounted on slides for storage at −80 C. All dissections were accomplished with the aid of a dissecting scope while maintaining freezing conditions. Only those sections exhibiting visual landmarks for VTA, as based on stereotaxic coordinates [29], were used. For the VTA, these coordinates ranged from −4.92 to −6.72 from bregma, resulting in approximately 6 sections per animal. The VTA dissections included a small portion of medial substantia nigra, and care was taken to exclude the medial interpeduncular nucleus (IPN) and any dorsal regions. All dissections per animal were pooled, and stored frozen for future use.

### Protein Processing

Samples were lysed by sonication in hot 1% SDS with phosphatase and protease inhibitors (Sigma P5725, P2850, P8340), subsequently boiled for 10 minutes, cooled, and then centrifuged at 10,000 g for 5 minutes to remove insoluble material. Supernatants were quantified for protein (DC Assay, Biorad) and frozen for future use.

### Western Blotting and Quantification

30 µg of protein lysate was loaded onto 4–12% gradient gels (NuPage, Invitrogen), separated by SDS-PAGE, transferred to nitrocellulose, and blocked with 5% non-fat milk prior to antibody incubation. Alexa Fluor 680 (Molecular Probes) and IRDye 800 (Rockland) Fluorescent secondary antibodies were detected by LI-COR Odyssey® Infrared Imaging System. Fluorescent densities were determined using Odyssey Software. Both phospho- and total signals were determined within the same band. Ratios of phosphorylated to non-phosphorylated proteins were calculated.

### Electrophysiology

Electrophysiology methods for dopamine whole-cell patch clamp recording, dopamine neuron identification, and leptin application were as previously described [4], [30], [31]. Male C57BL/6 mice aged 3 weeks were used. U0126, a specific MEK1/2 inhibitor [32] (10 µM), or vehicle (DMSO) was bath applied to the slices at least 20 minutes before the application of leptin, and remained present during and after leptin. All data were sampled at 3–10 kHz and filtered at 1–3 kHz with an Apple Macintosh computer using Axograph 4.9 (Axon Instruments). Electrophysiological data were analyzed with Axograph 4.9 (Axon Instruments) and plotted with Igor Pro software (WaveMetrics, Lake Oswego, Oregon).

### Statistical Analysis

Graphs shown are means of the grouped percentages ± SEM. Two-tailed unpaired Student's t-test was used to compare leptin and vehicle groups (for individual proteins), using GraphPad Prism. Two-way ANOVA was used to compare inhibitor versus leptin groups using the statistical package SPSS (SPSS Inc., Chicago, Il.).
